# Examining Associations Between Smartphone Use and Clinical Severity in Frontotemporal Dementia: Proof-of-Concept Study

**DOI:** 10.2196/52831

**Published:** 2024-06-26

**Authors:** Emily W Paolillo, Kaitlin B Casaletto, Annie L Clark, Jack C Taylor, Hilary W Heuer, Amy B Wise, Sreya Dhanam, Mark Sanderson-Cimino, Rowan Saloner, Joel H Kramer, John Kornak, Walter Kremers, Leah Forsberg, Brian Appleby, Ece Bayram, Andrea Bozoki, Danielle Brushaber, R Ryan Darby, Gregory S Day, Bradford C Dickerson, Kimiko Domoto-Reilly, Fanny Elahi, Julie A Fields, Nupur Ghoshal, Neill Graff-Radford, Matthew G H Hall, Lawrence S Honig, Edward D Huey, Maria I Lapid, Irene Litvan, Ian R Mackenzie, Joseph C Masdeu, Mario F Mendez, Carly Mester, Toji Miyagawa, Georges Naasan, Belen Pascual, Peter Pressman, Eliana Marisa Ramos, Katherine P Rankin, Jessica Rexach, Julio C Rojas, Lawren VandeVrede, Bonnie Wong, Zbigniew K Wszolek, Bradley F Boeve, Howard J Rosen, Adam L Boxer, Adam M Staffaroni, ALLFTD Consortium

**Affiliations:** 1 Department of Neurology, Memory and Aging Center, Weill Institute for Neurosciences University of California, San Francisco San Francisco, CA United States; 2 Department of Epidemiology and Biostatistics, University of California, San Francisco San Francisco, CA United States; 3 Department of Quantitative Health Sciences, Division of Clinical Trials and Biostatistics, Mayo Clinic Rochester, MN United States; 4 Department of Neurology, Mayo Clinic Rochester, MN United States; 5 Department of Neurology, Case Western Reserve University Cleveland, OH United States; 6 Department of Neurosciences, University of California, San Diego La Jolla, CA United States; 7 Department of Neurology, University of North Carolina Chapel Hill, NC United States; 8 Department of Neurology, Vanderbilt University Nashville, TN United States; 9 Department of Neurology, Mayo Clinic Jacksonville, FL United States; 10 Department of Neurology, Massachusetts General Hospital and Harvard Medical School Boston, MA United States; 11 Department of Neurology, University of Washington Seattle, WA United States; 12 Department of Neurology, The Deane Center for Wellness and Cognitive Health, Icahn School of Medicine at Mount Sinai New York, NY United States; 13 James J. Peters Veterans Affairs Medical Center New York, NY United States; 14 Department of Psychiatry and Psychology, Mayo Clinic Rochester, MN United States; 15 Department of Neurology, Knight Alzheimer's Disease Research Center, Washington University St. Louis, MO United States; 16 Department of Neurology, Columbia University New York, NY United States; 17 Department of Psychiatry and Human Behavior, Brown University Providence, RI United States; 18 Department of Pathology, University of British Columbia Vancouver, BC Canada; 19 Stanley H. Appel Department of Neurology, Nantz National Alzheimer Center, Houston Methodist Research Institute, Weill Cornell Medicine Houston, TX United States; 20 Department of Neurology, David Geffen School of Medicine, University of California, Los Angeles Los Angeles, CA United States; 21 Department of Neurology, Icahn School of Medicine at Mount Sinai New York, NY United States; 22 Department of Neurology, University of Colorado Aurora, CO United States; 23 Department of Psychiatry, Massachusetts General Hospital and Harvard Medical School Boston, MA United States; 24 see Acknowledgements San Francisco, CA United States

**Keywords:** digital, technology, remote, monitoring, cognition, neuropsychology, cognitive impairment, neurodegenerative, screening, clinical trials, mobile phone

## Abstract

**Background:**

Frontotemporal lobar degeneration (FTLD) is a leading cause of dementia in individuals aged <65 years. Several challenges to conducting in-person evaluations in FTLD illustrate an urgent need to develop remote, accessible, and low-burden assessment techniques. Studies of unobtrusive monitoring of at-home computer use in older adults with mild cognitive impairment show that declining function is reflected in reduced computer use; however, associations with smartphone use are unknown.

**Objective:**

This study aims to characterize daily trajectories in smartphone battery use, a proxy for smartphone use, and examine relationships with clinical indicators of severity in FTLD.

**Methods:**

Participants were 231 adults (mean age 52.5, SD 14.9 years; n=94, 40.7% men; n=223, 96.5% non-Hispanic White) enrolled in the Advancing Research and Treatment of Frontotemporal Lobar Degeneration (ARTFL study) and Longitudinal Evaluation of Familial Frontotemporal Dementia Subjects (LEFFTDS study) Longitudinal Frontotemporal Lobar Degeneration (ALLFTD) Mobile App study, including 49 (21.2%) with mild neurobehavioral changes and no functional impairment (ie, prodromal FTLD), 43 (18.6%) with neurobehavioral changes and functional impairment (ie, symptomatic FTLD), and 139 (60.2%) clinically normal adults, of whom 55 (39.6%) harbored heterozygous pathogenic or likely pathogenic variants in an autosomal dominant FTLD gene. Participants completed the Clinical Dementia Rating plus National Alzheimer’s Coordinating Center Frontotemporal Lobar Degeneration Behavior and Language Domains (CDR+NACC FTLD) scale, a neuropsychological battery; the Neuropsychiatric Inventory; and brain magnetic resonance imaging. The ALLFTD Mobile App was installed on participants’ smartphones for remote, passive, and continuous monitoring of smartphone use. Battery percentage was collected every 15 minutes over an average of 28 (SD 4.2; range 14-30) days. To determine whether temporal patterns of battery percentage varied as a function of disease severity, linear mixed effects models examined linear, quadratic, and cubic effects of the time of day and their interactions with each measure of disease severity on battery percentage. Models covaried for age, sex, smartphone type, and estimated smartphone age.

**Results:**

The CDR+NACC FTLD global score interacted with time on battery percentage such that participants with prodromal or symptomatic FTLD demonstrated less change in battery percentage throughout the day (a proxy for less smartphone use) than clinically normal participants (*P*<.001 in both cases). Additional models showed that worse performance in all cognitive domains assessed (ie, executive functioning, memory, language, and visuospatial skills), more neuropsychiatric symptoms, and smaller brain volumes also associated with less battery use throughout the day (*P*<.001 in all cases).

**Conclusions:**

These findings support a proof of concept that passively collected data about smartphone use behaviors associate with clinical impairment in FTLD. This work underscores the need for future studies to develop and validate passive digital markers sensitive to longitudinal clinical decline across neurodegenerative diseases, with potential to enhance real-world monitoring of neurobehavioral change.

## Introduction

### Background

Frontotemporal lobar degeneration (FTLD) is a common cause of dementia in individuals aged <65 years [1,2]. FTLD encompasses a group of neuropathologically distinct diseases that result in an overlapping set of dementia syndromes with heterogeneous symptoms, including those defined by primary behavior, language, or sensorimotor changes [3,4]. The timely detection of neurodegenerative diseases such as FTLD is a core public health strategy to reduce the individual, caregiver, and socioeconomic burden of dementia [5-7]. As we enter the era of disease-modifying treatments for neurodegenerative diseases, early detection is critical to identify those eligible for clinical trial participation and early treatment to slow or stop disease progression [8-10].

However, current assessment practices for detecting neurobehavioral changes associated with neurodegenerative disease are limited. In-person neuropsychological and neurological evaluations are the gold standard for determining the presence of cognitive impairment and identifying clinical phenotypes suggestive of an underlying neurodegenerative process; unfortunately, their high costs and restricted availability via specialty dementia clinics and research centers limit access for those with fewer financial resources and lower health literacy as well as those who reside in more remote geographic locations. In addition, evaluating a person at a single appointment provides only a *snapshot* of neurobehavioral functioning, which does not account for the dynamic nature of human behavior that fluctuates diurnally and is influenced by other dynamic factors (eg, sleep, fatigue, mood, and medications), limiting sensitivity for detecting early subtle declines [11,12]. Traditional neuropsychological assessment also lacks ecological validity because interpretations of functioning are based on task performance in a tightly controlled testing environment, which seldom reflects a patient’s typical daily experience.

Remote monitoring of health status and behavior through the use of digital health tools is a promising solution to overcome the numerous limitations of in-person assessment and has been identified as a priority by several leading health organizations, including the US Food and Drug Administration [13], the US Department of Health and Human Services [14], and the National Institutes of Health [15,16]. Passive digital monitoring in particular (ie, monitoring behavior passively and unobtrusively through remote sensors) represents a low-burden and highly scalable method for improved detection and monitoring of *real-world* neurobehavioral change in neurodegenerative disease. Naturalistic behavioral data collected via in-home remote sensors have shown sensitivity to clinical severity in Alzheimer disease [17-25]; for example, older adults with mild cognitive impairment exhibit significant declines in the number of days with computer use and daily time spent on the computer per day compared to those without cognitive impairment [18]. As an extension of this work, we aim to examine overall daily smartphone use and its association with clinical severity in FTLD. We focused on FTLD as a specific use case to study the construct of passively collected smartphone data in the context of a neurodegenerative disease that manifests with well-characterized neurobehavioral changes.

### Objectives

Thus, the aims of this study were to (1) examine passively collected battery percentage trajectories as a proxy for smartphone use throughout the day and (2) test associations between daily battery percentage trajectories and measures of cognitive and functional impairment and neurodegeneration in FTLD. Time-stamped battery percentage data can be easily accessed through public application programming interfaces (APIs) for both iOS and Android devices and have previously been associated with smartphone use [26-28]. Although smartphone screen time or app use time may be a more face-valid measure of smartphone use, access to these data has historically been restricted on iOS devices. This has been a major barrier to accessibility in passive monitoring research because nearly 30% of smartphone users worldwide have iOS devices [29]. Thus, it is worthwhile to examine battery percentage as a more accessible proxy for overall smartphone use. Consistent with prior research on computer use in older adults with cognitive impairment, we hypothesized that individuals with greater FTLD overall disease severity (ie, more severe functional impairment, worse cognitive performance, greater neuropsychiatric symptoms, and more brain atrophy) would demonstrate lower levels of daily smartphone use.

## Methods

### Participants

Participants were enrolled in the ARTFL (Advancing Research and Treatment of Frontotemporal Lobar Degeneration) study and LEFFTDS (Longitudinal Evaluation of Familial Frontotemporal Dementia Subjects) Longitudinal Frontotemporal Lobar Degeneration (ALLFTD) Mobile App study through the multisite ALLFTD (NCT04363684) study and University of California San Francisco studies of FTLD (AG038791, AG062422, and AG019724), as described previously [30]. The participants were those who had a referring diagnosis of an FTLD clinical syndrome or those who were members of a family with a strong family history of an FTLD syndrome. Additional inclusion criteria were as follows: (1) aged ≥18 years, (2) access to a smartphone, and (3) English reported as the primary language. Participants were asked to use their own smartphones. Recruitment primarily targeted those with Clinical Dementia Rating Dementia Staging Instrument plus National Alzheimer’s Coordinating Center Frontotemporal Lobar Degeneration Behavior and Language Domains (CDR+NACC FTLD) global scores of <2, but participants who were more severely impaired were not excluded. Data for this study were collected from August 2020 to April 2023. During this period, 257 participants were enrolled and logged into the ALLFTD Mobile App on their personal smartphones. Participants were only included in this secondary analysis of the ALLFTD Mobile App study if they had at least 14 continuous days of passive smartphone monitoring data, consistent with prior digital phenotyping studies attempting to capture typical daily behavior [31]. Thus, of the initial 257 participants, 231 (89.9%) were included in the final sample after 26 (10.1%) participants were excluded because they first logged in <14 days before the date on which these data were pulled in April 2023. Of these 231 participants, 92 (39.8%) were classified as having neurobehavioral symptoms at the prodromal stage (ie, no functional impairment) or fully symptomatic (ie, with functional impairment) level of severity that are consistent with an FTLD-related clinical phenotype per conference consensus with neurologists and neuropsychologists following published criteria [32-35]. Participants who were symptomatic had either sporadic FTLD or a confirmed pathogenic or likely pathogenic variant in an autosomal dominant FTLD gene (ie, a pathogenic expansion in the chromosome 9 open reading frame 72 [C9orf72] gene or a known pathogenic or likely pathogenic variant in the progranulin (GRN) or microtubule-associated protein tau [MAPT] genes; conducted as described previously [36]). The remaining participants (139/231, 60.2%) were asymptomatic clinically normal family members of the prodromal or symptomatic individuals who (1) carried a pathogenic or likely pathogenic FTLD gene variant (55/139, 39.6%), (2) tested negative for known pathogenic or likely pathogenic FTLD variants (50/139, 36%), or (3) did not yet have results available from genetic testing (34/139, 24.5%).

### Ethical Considerations

The study was approved by a centralized single institutional review board at Johns Hopkins Medicine (IRB # 20-29891), and all participants provided written informed consent.

### Measures

#### Passively Monitored Smartphone Battery Percentage

We used the first 30 days of participants’ smartphone data for this study with the goal of understanding whether approximately 1 month of smartphone monitoring could reflect baseline neurobehavioral status without capturing longitudinal disease-related decline [18,37]. Participants downloaded the ALLFTD Mobile App onto their personal smartphones. The app is designed to deliver both active mobile cognitive assessments and passively collect smartphone use data [30], including battery percentage. The ALLFTD Mobile App was programmed to collect battery percentage every 15 minutes. Due to some variability around this timing in the actual data collected (ie, some missing data points and some data collected over shorter intervals), data were aggregated to reflect the average battery percentage per hour of each study day per participant. This resulted in a comparable number of data points per day across participants. The ALLFTD Mobile App also recorded information about participants’ smartphone model, which was used to estimate the age of the smartphone (ie, calculated on the basis of the smartphone model release date and the first date of participation in this study).

#### Functional, Cognitive, and Neuropsychiatric Assessment

All participants underwent comprehensive functional and cognitive assessment at a parent study visit at the beginning of their smartphone monitoring study period. Informant and participant interviews were conducted to characterize the level of cognitive and everyday functioning impairment using the CDR+NACC FTLD scale [38], which is a validated, modified version of the CDR [39] that has higher sensitivity to functional impairment in FTLD. CDR+NACC FTLD global scores [40] were used to categorize participants into disease severity groups: 0=unimpaired, 0.5=prodromal, and ≥1=symptomatic. Domain-specific cognitive functioning was assessed via a comprehensive battery of well-validated neuropsychological tests. The previously published Uniform Data Set (Version 3) Executive Function composite score was used as our measure of executive functioning, comprising Trail Making Test A and B, phonemic fluency (generating words beginning with F and L), number span backwards, and category fluency (animals and vegetables) [41,42]. Sample-based *z* scores were calculated for indices of memory, including immediate and delayed free recall on the California Verbal Learning Test-3 Brief Form [43], as well as Benson Complex Figure Delayed Recall [44,45]. A composite memory *z* score was created by taking the mean of the *z* scores across these memory tests. Language functioning was assessed via the Multilingual Naming Test [46]. Visuospatial functioning was assessed via the Benson Complex Figure Copy [45]. Informants also completed the Neuropsychiatric Inventory [47] to assess the presence and severity of neuropsychiatric symptoms in participants.

#### Neuroimaging

Of the 231 participants, a subset (n=189, 81.8%) completed neuroimaging. Participants were scanned on 3 Tesla magnetic resonance imaging (MRI) scanners. T1-weighted images were acquired as magnetization-prepared rapid gradient echo images using the following parameters: 240×256×256 matrix; approximately 170 slices; voxel size=1.05×1.05×1.25 mm^3^; and flip angle, echo time, and repetition time varied by vendor. A standard imaging protocol was used across all centers, and all images were reviewed for quality by a core group at the Mayo Clinic, Rochester, Minnesota, United States. Details of image acquisition, processing, and harmonization have been published elsewhere [48]. Total gray matter volume was used as the primary neuroimaging variable of interest. Total intracranial volumes were regressed out (using a simple linear regression with gray matter volume as outcome and total intracranial volume as the only predictor) before inclusion in analyses to account for interindividual volumetric differences in head size on gray matter volume.

### Statistical Analyses

Differences in demographic and clinical characteristics across the CDR+NACC FTLD–defined disease severity groups were tested with 1-way ANOVA and chi-square tests for continuous variables and categorical variables, respectively. Raw battery percentage data were plotted against the time of day to inform statistical analysis. Linear mixed effects (LME) regression models were then used to model the linear, quadratic, and cubic effects of time (ie, hour of the day; 0=midnight; 23=11 PM) on battery percentage. Person-specific random intercepts and random effects of time (linear, quadratic, and cubic) were modeled. To determine whether daily patterns of battery percentage trajectories (ie, a proxy for smartphone use) varied as a function of FTLD disease severity, LME models examined linear, quadratic, and cubic effects of time and their interaction with each measure of disease severity separately (ie, CDR+NACC FTLD group, cognitive domain *z* scores, neuropsychiatric symptoms, and whole brain gray matter volumes). All LME models covaried for age, sex, smartphone type (iOS vs Android), and estimated smartphone age (calculated on the basis of the smartphone model release date and the first date of participation in this study). A post hoc sensitivity analysis was conducted in a subset of participants (162/231, 70.1%) whose age range was matched across the CDR+NACC FTLD groups. To understand whether subtle differences in neurobehavioral functioning related to daily smartphone battery use trajectories in an *unimpaired* sample, we conducted additional sensitivity analyses, which repeated all models in the subset of participants who were clinically normal (139/231, 60.2%). Regression estimates are reported as standardized betas, which represent the predicted change in the outcome as a function of each predictor in units of SDs. All analyses were performed using R (version 4.2.0; R Foundation for Statistical Computing). The *lme4* package was used to conduct the LME regressions [49].

## Results

### Participant Characteristics

Table 1 shows demographic and clinical characteristics by disease severity group. Participants had a mean age of 52.5 (SD 14.9) years and a mean of 16 (SD 2.2) years of education. Of the 231 participants, 94 (40.7%) were men, and 223 (96.5%) identified as non-Hispanic White. Nearly three-fourths of the participants (171/231, 74%) had results of genetic testing available, of whom 45% (77/171) had heterozygous pathogenic or likely pathogenic variants in an FTLD gene. Clinically normal participants were statistically significantly younger (*P*<.001) and more likely to be women (*P*<.001) than those with prodromal or symptomatic FTLD, consistent with the larger parent study samples (ALLFTD Mobile App study [30]; ALLFTD [40,50]). There were no other clear imbalances in other demographic and clinical characteristics across the 3 groups. Overall, participants had a mean of 28.3 (SD 4.19; range 14-30) days of smartphone monitoring data. On average, participants’ smartphones were 2.8 (SD 1.53; range 0-7) years old.

**Table 1:** Participant characteristics by disease severity group (n=231).

								A (clinically normal; n=139)			B (prodromal; n=49)			C (symptomatic; n=43)			*P* value				Pairwise comparisons^a^		
**Demographics**																							
	Age (years), mean (SD)						46.3 (13.9)			59.7 (12.1)			64.3 (9.3)			<.001				A<B, C			
	Sex (male), n (%)						41 (29.5)			30 (61.2)			23 (53.5)			<.001				A<B, C			
	Education (years), mean (SD)						16.3 (2.1)			16.5 (2.4)			16.7 (2.4)			.61				N/A^b^			
	Race and ethnicity (non-Hispanic White), n (%)						134 (96.4)			48 (98)			41 (95.3)			.79				N/A			
**Study characteristics**																							N/A
	Total study days, mean (SD)						28.3 (4.2)			28.1 (4.3)			28.6 (4.2)			.88							
	**Smartphone type, n (%)**																	.42					
		iOS					97 (69.8)			37 (75.5)			27 (62.8)										
		Android					42 (30.2)			12 (24.5)			16 (37.2)										
	Estimated smartphone age (years), mean (SD)						2.7 (1.5)			3.0 (1.5)			2.9 (1.6)			.30							
	**Genetic status**																						
		**Genetic testing results, n (%)**																.56					
			Not available				34 (24)			13 (26.5)			13 (30.2)										
			**Available**				105 (75.5)			36 (73.5)			30 (69.8)										
				**Mutation carrier**			55 (52.4)			15 (41.7)			7 (23.3)										
					C9orf72^c^	29 (52.7)			8 (53.3)			3 (42.9)											
					GRN^d^	7 (12.7)			1 (6.7)			0 (0)											
					MAPT^e^	16 (29.1)			6 (40)			3 (42.9)											
					Other^f^	3 (5.5)			0 (0)			1 (14.3)											
	**Clinical phenotype**																	N/A					
		Mild cognitive impairment^g^					N/A			39 (79.6)			N/A										
		bvFTD^h^					N/A			N/A			25 (58.1)										
		svPPA^i^					N/A			N/A			6 (14)										
		nfvPPA^j^					N/A			N/A			3 (7)										
		lvPPA^k^					N/A			N/A			1 (2.3)										
		PSP-RS^l^					N/A			3 (6.1)			4 (9.3)										
		CBS^m^					N/A			2 (4.1)			2 (4.7)										
		Other^n^					N/A			5 (10.2)			2 (4.7)										

^a^Pairwise comparisons were evaluated with the Tukey honestly significant difference test.

^b^N/A: not applicable.

^c^C9orf72: chromosome 9 open reading frame 72.

^d^GRN: progranulin.

^e^MAPT: microtubule-associated protein tau.

^f^Identified pathogenic or likely pathogenic variants in genes less commonly identified as genetic causes of frontotemporal lobar degeneration (FTLD; ie, other than C9orf72, GRN, or MAPT). The specific genetic variant is not provided to protect participant anonymity.

^g^Includes behavior-, cognitive-, and language-predominant mild cognitive impairment syndromes.

^h^bvFTD: behavioral variant frontotemporal dementia.

^i^svPPA: semantic variant primary progressive aphasia.

^j^nfvPPA: nonfluent variant primary progressive aphasia.

^k^lvPPA: logopenic variant primary progressive aphasia.

^l^PSP-RS: progressive supranuclear palsy–Richardson syndrome.

^m^CBS: corticobasal syndrome.

^n^Includes FTLD-amyotrophic lateral sclerosis or a change in neurobehavior that may not meet full diagnostic criteria for any particular FTLD syndrome.

### Daily Smartphone Battery Percentage

**Figure 1:**
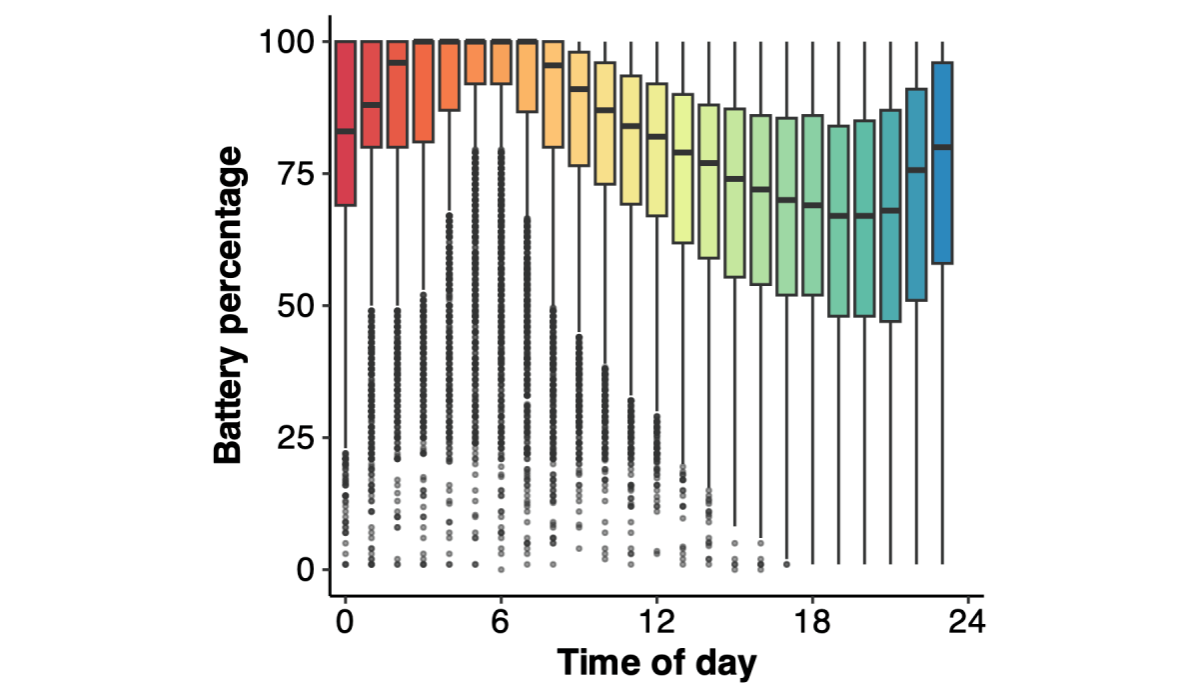
Visualization of raw battery percentage data for all participants binned by time of day (0=midnight; 23=11 PM).

Visualization of the raw battery percentage data by the time of day (Figure 1) shows a nonlinear trajectory such that, on average, battery percentage increased from midnight to approximately 6 AM, then decreased until about 7 PM, and then increased again through 11 PM. These temporal patterns presumably represent typical patterns of charging and charge use of the smartphone throughout the day. Multimedia Appendix 1 presents raw battery percentage data by disease severity group. The shape of these raw data motivated consideration of a cubic model. Thus, we first tested the fit of the LME regression modeling the linear, quadratic, and cubic effects of the time of day on battery percentage, covarying for age, sex, smartphone type, and estimated smartphone age. The cubic model’s conditional pseudo-*R*^2^ (ie, the proportion of variance explained by both fixed and random factors) was 0.37. The likelihood ratio tests indicated that the full cubic model had statistically significantly better fit than LME regressions modeling only the linear (*χ*^2^_2_=4283.6; *P*<.001) and quadratic (*χ*^2^_1_=4118.8; *P*<.001) effects of time.

**Figure 2:**
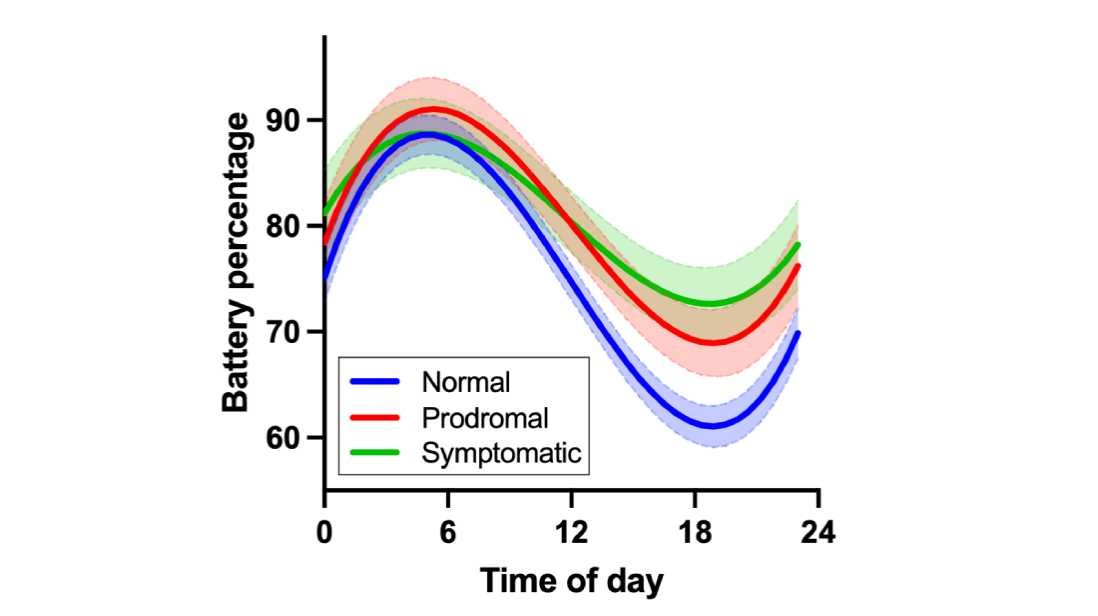
Participants with prodromal frontotemporal lobar degeneration (FTLD) and those with symptomatic FTLD had flatter battery curves throughout the day (ie, a proxy for less smartphone use) than clinically normal participants on average. Error bands represent pointwise 95% CIs.

LME regression indicated that the interactions between disease severity group and the linear, quadratic, and cubic effects of the time of day were associated with battery percentage (Table 2). Visualization of model results suggests that participants with prodromal FTLD and those with symptomatic FTLD had *flatter* battery curves throughout the day (ie, shallower decreases from maximum to minimum battery percentage as well as a higher minimum battery percentage; a proxy for less smartphone use) than clinically normal participants on average (Figure 2). Examination of pairwise disease severity group contrasts showed that participants with symptomatic FTLD also had significantly less battery use than participants with prodromal FTLD (*P*=.003 or *P*<.001 in all cases).

**Table 2:** Linear mixed effects regression results showing significant relationships between disease severity groups and battery percentage trajectories throughout the day.

	β (95% CI)	*P* value
Baseline age	.06 (.00 to .13)	.047
Sex (reference: female)	−.03 (−.14 to .08)	.62
Smartphone type (reference: Android)	.01 (−.11 to .12)	.89
Estimated smartphone age	−.04 (−.08 to −.01)	.03
Time of day (linear)	1.74 (1.66 to 1.83)	<.001
Time of day (quadratic)	−5.27 (−5.45 to −5.09)	<.001
Time of day (cubic)	3.28 (3.17 to 3.40)	<.001
Prodromal (reference: normal)	.24 (.10 to .39)	.001
Symptomatic (reference: normal)	.27 (.11 to .43)	.001
Time of day (linear)×prodromal	−.23 (−.40 to −.07)	.006
Time of day (linear)×symptomatic	−.56 (−.73 to −.38)	<.001
Time of day (quadratic)×prodromal	.88 (.53 to 1.24)	<.001
Time of day (quadratic)×symptomatic	1.65 (1.29 to 2.01)	<.001
Time of day (cubic)×prodromal	−.58 (−.81 to −.36)	<.001
Time of day (cubic)×symptomatic	−.99 (−1.22 to −.76)	<.001

**Figure 3:**
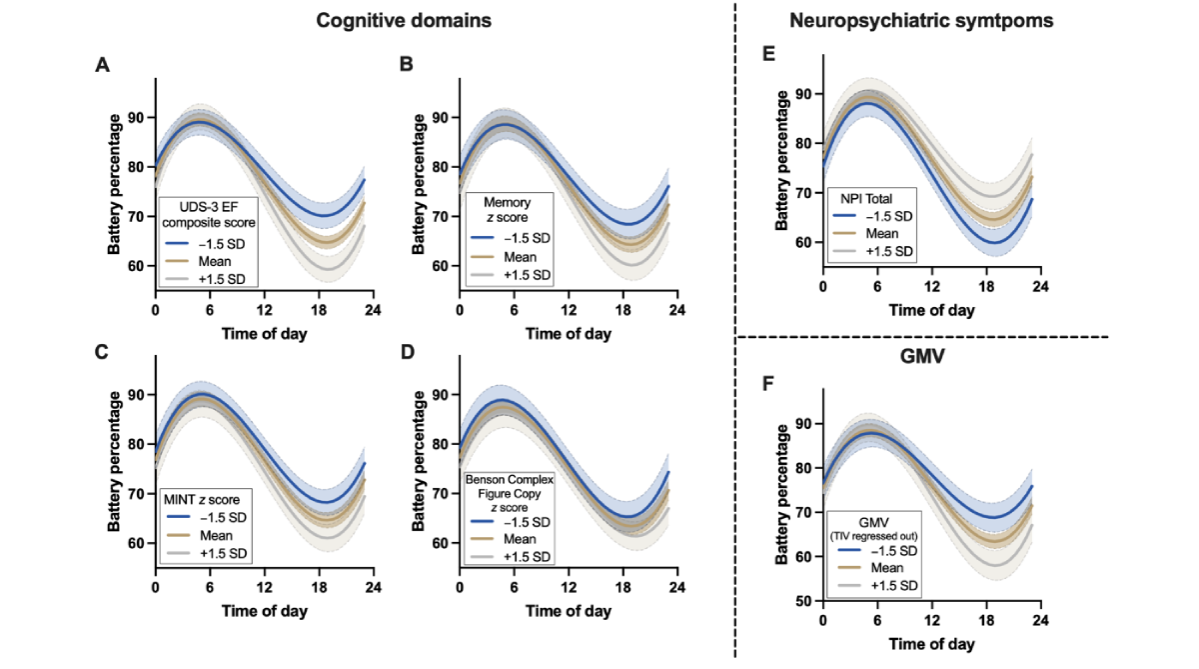
Daily battery percentage trajectories were significantly moderated by (A) executive functioning, (B) memory, (C) language, (D) visuospatial skills, (E) neuropsychiatric symptoms, and (F) total gray matter volumes. Participants with worse neurobehavioral outcomes had smaller daily decreases from peak to minimum battery percentage on average, suggesting less smartphone use throughout the day. GMV: gray matter volume; MINT: Multilingual Naming Test; NPI: Neuropsychiatric Inventory; TIV: total intracranial volume; UDS3-EF: Uniform Data Set (Version 3) Executive Function.

Similar patterns emerged when examining all other indices of clinical severity. Each cognitive domain statistically significantly moderated the relationship between the time of day and battery percentage such that participants with worse cognitive functioning had flatter battery curves throughout the day, suggesting less smartphone use (Table 3 [executive functioning, memory, language, and visuospatial skills]; Figures 3A-3D). Neuropsychiatric symptom severity also moderated the relationship between the time of day and battery percentage such that participants with higher neuropsychiatric symptom ratings had flatter battery curves throughout the day, suggesting less smartphone use (Table 3 [neuropsychiatric symptoms]; Figure 3E). Examination of each Neuropsychiatric Inventory item (*yes* or *no*) in separate LME models suggested that participants with agitation, depression, apathy, disinhibition, irritability, motor disturbance, nighttime behaviors, and changes in appetite had less smartphone use (Table 4). Delusions, hallucinations, anxiety, and elation did not statistically significantly relate to battery use trajectories throughout the day (Table 4). Finally, total gray matter volume also moderated the relationship between the time of day and battery percentage such that participants with smaller gray matter volumes had flatter battery curves throughout the day, suggesting less smartphone use (Table 3 [gray matter volume]; Figure 3F). Of all indices of clinical severity presented in Table 3, executive functioning and total gray matter volume appeared to have the largest effect sizes on smartphone battery trajectories.

Given the age difference across disease severity groups, we repeated the first LME model examining battery percentage trajectories by CDR+NACC FTLD group after restricting the age range of the clinically normal group to be identical to that of the group with prodromal FTLD and the group with symptomatic FTLD (participants aged 44-81 years in all groups; clinically normal: 70/139, 50.4%). The interactions between disease severity group and the linear, quadratic, and cubic effects of the time of day on battery percentage are fairly consistent, showing that the participants who were symptomatic had lower battery use than clinically normal participants (interaction with linear time: β=−.23, 95% CI −.40 to −.06; *P*=.009; interaction with quadratic time: β=.65, 95% CI .23 to 1.07; *P*=.003; interaction with cubic time: β=−.39, 95% CI −.66 to −.12; *P*=.004). However, the difference between the prodromal and clinically normal participants no longer reached statistical significance (interaction with linear time: β=−.05, 95% CI −.13 to .22; *P*=.586; interaction with quadratic time: β=−.07, 95% CI −.50 to .36; *P*=.76; interaction with cubic time: β=.05, 95% CI −.22 to .33; *P*=.70).

**Table 3:** Results of separate linear mixed effects regression models showing significant relationships between battery percentage trajectories throughout the day and executive functioning, memory, language, visuospatial skills, neuropsychiatric symptoms, and whole brain gray matter volume (lower order terms and covariates are not displayed).

			β (95% CI)		*P* value
**Executive functioning**					
	Time of day (linear)×UDS3-EF^a^ composite score	.24 (.17 to .31)		<.001	
	Time of day (quadratic)×UDS3-EF composite score	−.65 (−.80 to −.50)		<.001	
	Time of day (cubic)×UDS3-EF composite score	.38 (.28 to .47)		<.001	
**Memory**					
	Time of day (linear)×memory *z* score	.21 (.13 to .28)		<.001	
	Time of day (quadratic)×memory *z* score	−.58 (−.74 to −.42)		<.001	
	Time of day (cubic)×memory *z* score	.34 (.24 to .44)		<.001	
**Language**					
	Time of day (linear)×MINT^b^ *z* score	.07 (.00 to .15)		.05	
	Time of day (quadratic)×MINT *z* score	−.24 (−.40 to −.08)		.002	
	Time of day (cubic)×MINT *z* score	.15 (.05 to .25)		.004	
**Visuospatial skills**					
	Time of day (linear)×Benson Complex Figure Copy *z* score	.10 (.04 to .16)		.001	
	Time of day (quadratic)×Benson Complex Figure Copy *z* score	−.28 (−.38 to −.19)		<.001	
	Time of day (cubic)×Benson Complex Figure Copy *z* score	.19 (.13 to .25)		<.001	
**Neuropsychiatric symptoms**					
	Time of day (linear)×NPI^c^ total score	−.06 (−.13 to .01)		.08	
	Time of day (quadratic)×NPI total score	.25 (.10 to .39)		.001	
	Time of day (cubic)×NPI total score	−.15 (−.25 to −.05)		.002	
**Gray matter volume (n=189)**					
	Time of day (linear)×gray matter volume	.22 (.14 to .29)		<.001	
	Time of day (quadratic)×gray matter volume	−.73 (−.88 to −.57)		<.001	
	Time of day (cubic)×gray matter volume	.45 (.35 to .55)		<.001	

^a^UDS3-EF: Uniform Data Set (Version 3) Executive Function.

^b^MINT: Multilingual Naming Test.

^c^NPI: Neuropsychiatric Inventory.

**Table 4:** Results of separate linear mixed effects regression models examining relationships between battery percentage trajectories throughout the day and each neuropsychiatric symptom (NPS) captured on the Neuropsychiatric Inventory. Models covaried for age, sex, smartphone type, and estimated smartphone age.

	Predictors modeling interactions with the time of day		
	NPS×time of day (linear), β (SE)	NPS×time of day (quadratic), β (SE)	NPS×time of day (cubic), β (SE)
Delusions	−.12 (.25)	−.25 (.53)	.43 (.34)
Hallucinations	−.61 (.40)	1.62 (.87)	−.78 (.55)
Agitation	−.36 (.10)^a^	1.06 (.20)^a^	−.65 (.13)^a^
Depression	−.34 (.08)^a^	.92 (.17)^a^	−.64 (.11)^a^
Anxiety	.05 (.08)	−.20 (.17)	.15 (.11)
Elation	.12 (.13)	−.38 (.26)	.29 (.17)
Apathy	−.43 (.08)^a^	1.32 (.17)^a^	−.81 (.11)^a^
Disinhibition	−.21 (.09)^a^	1.14 (.18)^a^	−.86 (.12)^a^
Irritability	−.20 (.07)^a^	.78 (.16)^a^	−.49 (.10)^a^
Motor disturbance	−.44 (.11)^a^	1.18 (.24)^a^	−.69 (.15)^a^
Nighttime behaviors	−.41 (.10)^a^	1.20 (.22)^a^	−.74 (.14)^a^
Changes in appetite	−.16 (.09)	.86 (.20)^a^	−.61 (.13)^a^

^a^*P* values met the threshold for significance.

Sensitivity analyses conducted among the 139 clinically normal participants showed that the following neurobehavioral measures were associated with daily battery percentage trajectories: executive functioning (interaction with linear time: β=.21, 95% CI .12 to .30; *P*<.001; interaction with quadratic time: β=−.57, 95% CI −.76 to −.38; *P*<.001; interaction with cubic time: β=.34, 95% CI .21 to .46; *P*<.001); memory (interaction with linear time: β=.33, 95% CI .24 to .42; *P*<.001; interaction with quadratic time: β=−.83, 95% CI −1.02 to −.63; *P*<.001; interaction with cubic time: β=.47, 95% CI .35 to .60; *P*<.001), and total gray matter volume (interaction with linear time: β=.32, 95% CI .22 to .41; *P*<.001; interaction with quadratic time: β=−.84, 95% CI −1.03 to −.64; *P*<.001; interaction with cubic time: β=.48, 95% CI .36 to .61; *P*<.001). Language, visuospatial functioning, and neuropsychiatric symptoms did not strongly associate with daily battery percentage trajectories in clinically normal participants. The directions of associations in these clinically normal participants were consistent with relationships described in the entire sample.

## Discussion

### Principal Findings

This study is the first to our knowledge to examine passively collected smartphone use data in a sample with neurodegenerative disease. The results highlight an accessible, low-burden, and scalable remote monitoring method that captured behaviors associated with cognitive, neuropsychiatric, and brain health outcomes in a sample of participants with FTLD. The findings support a proof of concept that this passive digital monitoring approach, in combination with other methods, warrants further evaluation as a potential tool to augment screening and monitoring neurobehavioral change in clinical populations. Consistent with our hypotheses, we found that daily trajectories of smartphone battery use (a proxy for overall smartphone use) were associated with gold standard measures of clinical severity in FTLD such that those with more severe levels of impairment had less smartphone use throughout the day. Relationships between battery percentage trajectories and executive functioning, memory, and gray matter volume also held in the subset of clinically normal participants, suggesting potential sensitivity to subclinical neurobehavioral differences.

### Comparison to Prior Work

These findings are consistent with previous studies showing that older adults with cognitive impairment have greater declines in everyday technology use compared to cognitively unimpaired older adults [18,51,52]. Other studies have shown that older adults with cognitive impairment report more difficulties using technology, representing a potential barrier to technology use [53,54]. Notably, the observed associations between battery percentage trajectories and cognitive functioning were not specific to particular cognitive domains, suggesting that the metrics of overall smartphone use may reflect a global transdiagnostic marker of functioning rather than a phenotype-specific marker (eg, executive functioning–predominant or language-predominant dysfunction). Thus, our findings may also not be specific to FTLD, and future work is needed to replicate findings in other populations with neurologic conditions. The use of a smartphone, like the use of a computer [18], is a cognitively complex task requiring the resources of many functions (eg, attention, executive function, working memory, and fine motor skill). As such, smartphone use patterns may be a particularly sensitive marker of early and subtle neurobehavioral change; however, additional research examining longitudinal changes in smartphone use over time is needed to support this hypothesis.

While this is the first study to our knowledge to report on passively collected smartphone use data in the context of neurodegenerative disease, there is a growing body of literature examining other passive streams of smartphone data as potential markers of neurobehavioral function in older adults; for example, passively collected data from smartphone accelerometers, GPS location, and touchscreen typing have been associated with symptom and disease severity in Parkinson disease, multiple sclerosis, and amyotrophic lateral sclerosis [55-64]. Future work should incorporate multiple passive smartphone data types for more comprehensive digital phenotyping and potentially improved clinical relevance in monitoring neurodegenerative disease.

Regarding the more technological aspects of passive smartphone data collection, previous studies have also reported similar variability around the frequency and timing of data collected per person. These studies have identified a number of factors that influence the collection and transfer of smartphone data to secure cloud-based servers, including smartphone hardware, data permissions, app engagement, wireless service, capacity of local data storage, data transmission limits, and even sociodemographic factors [65-67]. This has also been reported in other devices as well, including wearables [68-70]. Thus, thorough data cleaning is necessary to ensure that enough data points are captured to accurately represent activity for a given time period, as has been described previously [71,72].

Visualizing the raw battery percentage data was important for understanding daily patterns and supported the utility of daily smartphone battery percentage trajectories as a proxy for smartphone use. Average patterns in battery percentage appeared to track with typical diurnal sleep-wake rhythms: percentages increased up to morning (when mobile phones are likely charging) and decreased throughout the day (when participants were presumably awake and using their smartphones) until nighttime when percentages began to increase again. Careful examination of these raw data patterns, alongside measures of clinical severity, may support the development of specific metrics using battery percentage data that can be easily tracked over longer periods of time (eg, total battery drainage per day). Future work in this field should also consider examining the frequency and timing of smartphone battery charging as a way to track routine daily use patterns that may be clinically relevant. Tracking these metrics over many months or years would allow for future studies to examine person-specific changes in battery use over time and test longitudinal associations with neurodegenerative disease progression.

### Strengths and Limitations

The strengths of this study include a large, extensively characterized cohort with FTLD; the reporting of novel smartphone use data; and the use of passive digital monitoring techniques. However, we also acknowledge several limitations. First, there are certainly caveats to our approach using battery percentage as a proxy for smartphone use, including factors that are difficult to quantify and adjust for, such as the impact of hardware, software, and service connection on battery life [73]. In addition, the ALLFTD Mobile App does not record when a smartphone may have been plugged in for charging, potentially preventing periods of battery decline even when the smartphone may have been in use. Even so, the robust relationships observed with gold standard measures of functional impairment, cognition, neuropsychiatric symptoms, and brain volumes are encouraging. Second, it is likely that the mobile cognitive testing sessions administered by the ALLFTD Mobile App contributed to some smartphone battery use and that battery use may subsequently be affected by adherence to the mobile cognitive testing protocol. However, we have previously reported on adherence to mobile cognitive testing through this app [30], which showed that cognitive testing completion rates among asymptomatic participants, participants with prodromal FTLD, and participants with symptomatic FTLD were 71.4%, 78.4%, and 59%, respectively. These adherence rates do not match the stairstep effect of battery use reported in this study whereby cognitively normal participants exhibited the highest smartphone battery use, participants with prodromal FTLD demonstrated intermediate smartphone battery use, and participants with symptomatic FTLD showed the lowest smartphone battery use, suggesting that our results are not simply driven by adherence to the mobile cognitive testing protocol. Third, although we importantly controlled for the effects of age, sex, smartphone type, and estimated smartphone age in our statistical analyses, further replication is needed in samples whose disease severity groups are demographically matched. Fourth, it is possible that a 30-day monitoring period may not be enough time to most accurately capture routine smartphone use behavior. Future studies are needed to evaluate the psychometrics of passive smartphone use metrics across different periods of time to identify optimal lengths of follow-up. Fifth, our sample was also limited in demographic diversity because participants mostly identified as non-Hispanic White and were highly educated, reflective of the cohort in the ALLFTD study [10]. This is a crucial consideration when examining new tools that require access to technology because the implementation of such digital tools may inadvertently increase disparities among those with fewer resources to obtain technology that meets required software specifications. However, with the steadily increasing rates of smartphone ownership worldwide in addition to the implementation of government-funded programs to provide access to technology (eg, the Lifeline Program or “Obama Phone” [74]), there is growing consensus that smartphone monitoring could become universally accessible.

### Conclusions

In sum, our novel results demonstrate the feasibility of continuous, unobtrusive smartphone use monitoring, while also showing that smartphone use relates to the severity of neurobehavioral impairment in a sample with FTLD. We highlight these results as proof of concept because we believe that they support future research examining whether specific smartphone use metrics are clinically relevant and may have utility for monitoring clinical disease progression in FTLD and other neurodegenerative diseases. With continued validation, such passive monitoring methodologies for real-time, real-world, and remote monitoring have the potential to improve the monitoring of clinically meaningful neurobehavioral changes in individuals at risk for dementia.

## Appendix

Multimedia Appendix 1 Visualization of raw battery percentage data binned by time of day (0=midnight; 23=11 PM) by disease severity group: (A) clinically normal, (B) prodromal frontotemporal lobar degeneration (FTLD), and (C) symptomatic FTLD.

## Abbreviations

ALLFTD: Advancing Research and Treatment of Frontotemporal Lobar Degeneration (ARTFL study) and Longitudinal Evaluation of Familial Frontotemporal Dementia Subjects (LEFFTDS study) Longitudinal Frontotemporal Lobar Degeneration
API: application programming interface
ARTFL: Advancing Research and Treatment of Frontotemporal Lobar Degeneration
C9orf72: chromosome 9 open reading frame 72
CDR+NACC FTLD: Clinical Dementia Rating Dementia Staging Instrument plus National Alzheimer’s Coordinating Center Frontotemporal Lobar Degeneration Behavior and Language Domains
FTLD: frontotemporal lobar degeneration
GRN: progranulin
LEFFTDS: Longitudinal Evaluation of Familial Frontotemporal Dementia Subjects
LME: linear mixed effects
MAPT: microtubule-associated protein tau
MRI: magnetic resonance imaging
