# Supplementary material for: Examining Associations Between Smartphone Use and Clinical Severity in Frontotemporal Dementia: Proof-of-Concept Study
Source: JMIR Aging. 2024 Jun 26;7:e52831. doi: 10.2196/52831 (PMC11237775; doi:10.2196/52831)
Supplement: Multimedia Appendix 1 [file aging_v7i1e52831_app1.docx]

**Supplementary Figure 1**. Visualization of raw battery percentage data binned by time of day (0 = 12AM; 23 = 11PM) by disease severity group: A) clinically normal; B) prodromal FTLD; and C) symptomatic FTLD.
